# Benchmarking global fisheries discards

**DOI:** 10.1038/s41598-020-71021-x

**Published:** 2020-08-20

**Authors:** E. Gilman, A. Perez Roda, T. Huntington, S. J. Kennelly, P. Suuronen, M. Chaloupka, P. A. H. Medley

**Affiliations:** 1 Pelagic Ecosystems Research Group, Honolulu, USA; 2 Food and Agriculture Organization of the United Nations, Rome, Italy; 3 Poseidon Aquatic Resources Management Ltd., Lymington, UK; 4 IC Independent Consulting, Sydney, Australia; 5 Natural Resources Institute Finland, Helsinki, Finland; 6 Ecological Modelling Services Pty Ltd and Marine Spatial Ecology Lab, University of Queensland, Brisbane, Australia

**Keywords:** Ecology, Biodiversity, Ocean sciences

## Abstract

Discarding by fisheries is one of the most wasteful human marine activities, yet we have few estimates of its scale. Reliable estimates of global discards are essential for sustainable fisheries management. Using United Nations Food and Agriculture Organization databases on country-specific landings, we estimated the discard rate and magnitude for global marine and estuarine capture fisheries using fishery-specific discard rates derived from direct observations and global gear-specific discard rates estimated within a Bayesian modelling framework. An estimated 9.1 million tonnes are discarded annually (95% uncertainty interval: 7–16 M t)—or 10.8% of the global catch (95% UI: 10–12%). Encouragingly, this is about half of the annual global discard rate estimated in the late 1980s. Trawl fisheries, especially demersal otter trawls, warrant intensified efforts to reduce discards. Periodic benchmarks of global discards are needed to assess the performance of reduction efforts.

## Introduction

Discarding by marine fisheries is one of the most wasteful human marine activities and can have profound socioeconomic and ecological effects^1,2^. Yet there is large uncertainty over the current magnitude of discards and how they have changed over time. International guidelines have called for the reduction of discards in order to contribute to achieving the United Nations’ Sustainable Development Goal 14—to conserve and sustainably use the oceans, seas and marine resources for sustainable development^3–5^.

Fishing pressure on marine ecosystems have conventionally been monitored using only estimates of the portion of the catch that fishers retain. But because discards, the proportion of the catch that is not retained, can be substantial, monitoring and accounting for discards enables a more comprehensive, albeit still incomplete, estimate of total fishing mortality^6,7^, supporting more robust stock assessments and improved fisheries management^8^.

In addition to being a waste of natural resources, discards can affect fisheries’ socioeconomic performance. Reduced recruitment to a fishery can occur when large numbers of juveniles of marketable species are captured and discarded^9^. Discards in one fishery can reduce the catch of target species and revenue in others^10^. It is also operationally inefficient for vessels to catch and handle organisms that will not be retained^4,11^. Reducing discards by avoiding capture if possible, and by increasing retention and utilization, incentivized through the development of new markets for species and sizes currently with low or no economic value, can reduce pressure on overexploited target stocks^11,12^.

Discards can also alter ecosystem structure and processes. For example, discards can alter scavengers’ foraging behavior, distribution, diet, competition amongst species and community composition^13^. Discarding can alter the distribution of biomass within and between ecosystems. Some direct ecological effects of discards are detrimental, such as reducing the fecundity of scavengers due to density-dependent effects, while others are positive, such as providing an important food subsidy for marine obligate and facultative scavengers^14–16^. Non-target bycatch, some of which is discarded, can include relatively vulnerable species with low reproduction rates and other ‘slow’ life history traits^17,18^.

Fishers discard catch in response to numerous and continuously changing factors, including market conditions (low or no economic value), regulations (e.g., quota or size restrictions) and the quality of the catch^19–22^. Fishers may conduct over-quota discarding, and may high-grade, discarding lower value catch to make room for more valuable catch in response to quotas, and near the end of a trip when space in the hold is limited^23^. Retention bans, such as for species of conservation concern, and quotas in multispecies fisheries, while intended to incentivize the use of more selective fishing methods and gear, may be a regulatory-driven cause of discarding^6,23^.

The Food and Agriculture Organization of the United Nations (FAO) periodically reports to the United Nations General Assembly on progress in implementing United Nations’ resolutions on fisheries, including provisions on monitoring discards. We present the findings of FAO’s third estimate of global discards in marine fisheries^24^. We also describe a new, open access FAO database on global fisheries discards^25^.

## Results

Of an annual mean global catch of 84.6 million tonnes (95% CI: 82.2–91.6) for the period 2010–2014, 10.8% (95% Bayes-derived highest posterior density interval (HDI): 10.1–11.5%) or 9.1 million tonnes (95% CI: 6.7–16.1) was discarded annually.

**Figure 1:**
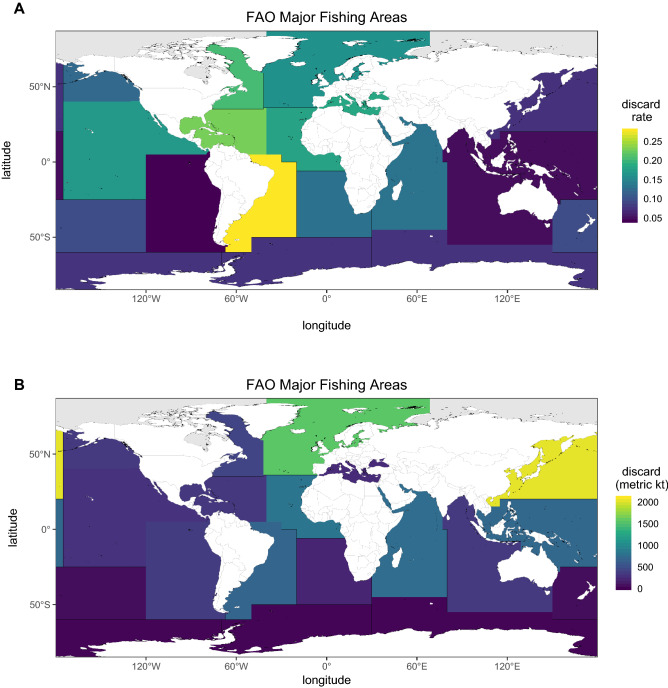
(**A**) Distribution of global fisheries discard rates in tonnes of discards per tonnes of total catch, and (**B**) discard levels in kilotonnes by FAO Major Fishing Area. Produced using the sf^36^ and colorspace^37^ packages for R. Reproduced with permission from Perez Roda^24^.

**Figure 2:**
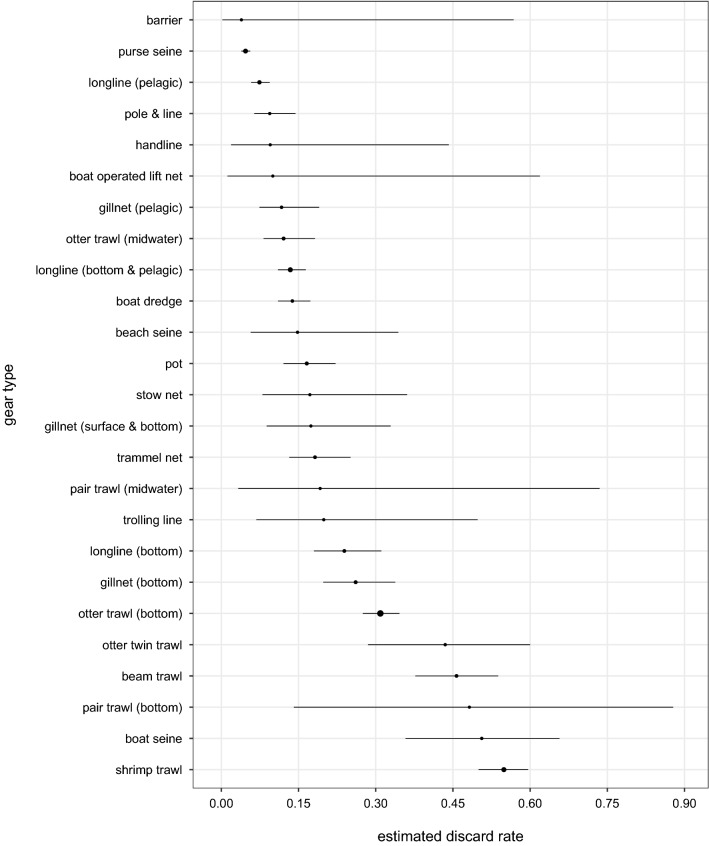
Mean gear-specific discard rates and 95% credible intervals, in tonnes of discards per tonnes of total catch. The size of the circle for the mean is proportional to the sample size. Reproduced with permission from Perez Roda^24^.

**Figure 3:**
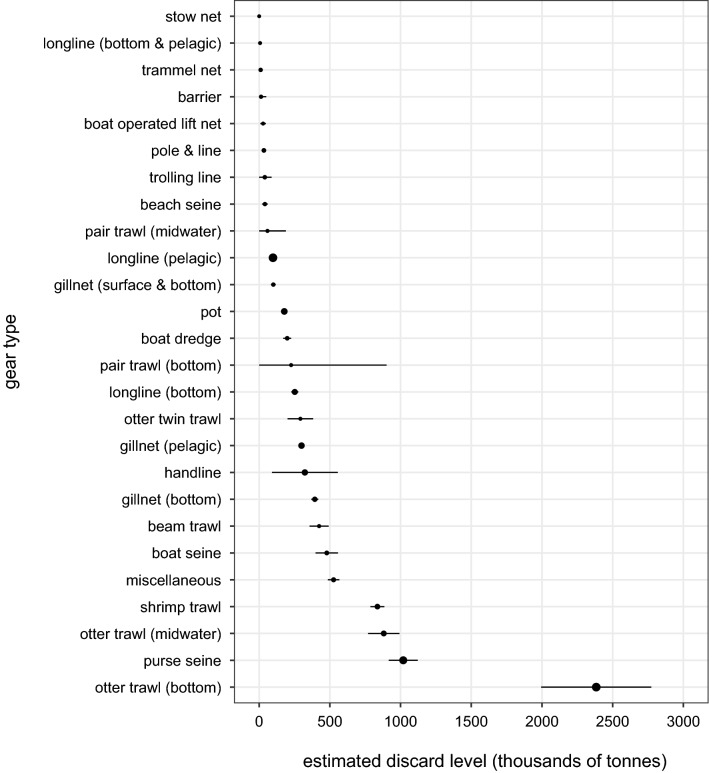
Mean gear-specific discard levels in kilotonnes and 95% CIs. The size of the circle for the mean is proportional to the sample size. Reproduced with permission from Perez Roda^24^.

Figures 1, 2, 3 and 5 are reproduced with permission from Perez Roda^24^. Figure 1 shows the spatial distribution of discard rates (weight of discards to weight of total catch) and levels (magnitude, in kilotonnes) by FAO fishing area. Regional discard rates ranged from 4% (95% HDI: 1.8–6.0, southeast Pacific) to over 29% (95% HDI: 26.7–28.4, southwest Atlantic). Some regions with relatively high discard rates had relatively low discard levels, and vice versa. For instance, the southwest Atlantic had the highest discard rate while contributing less than 8% of global discards. The northwest Pacific had the highest quantity of discards, contributing 22% of global levels, produced the highest landed catch (> 20 million t, 95% CI: 1.8–2.2 million t), and had the fifth lowest discard rate.

Figures 2 and 3 present gear-specific mean discard rates and levels, respectively. Discard rates ranged from 4% (95% CI: 0.2–56.8%) for combined barriers, fences and traps to 55% (95% CI: 50.0–59.6%) for shrimp trawls. Almost 60% of total annual discards were from combined trawl fisheries. Bottom otter trawl fisheries alone contributed 2.4 million t (95% CI: 2.0–2.8 million t) of discards—over a quarter of global discards. While purse seine fisheries had the second highest discard level, it had the second lowest discard rate.

**Figure 4:**
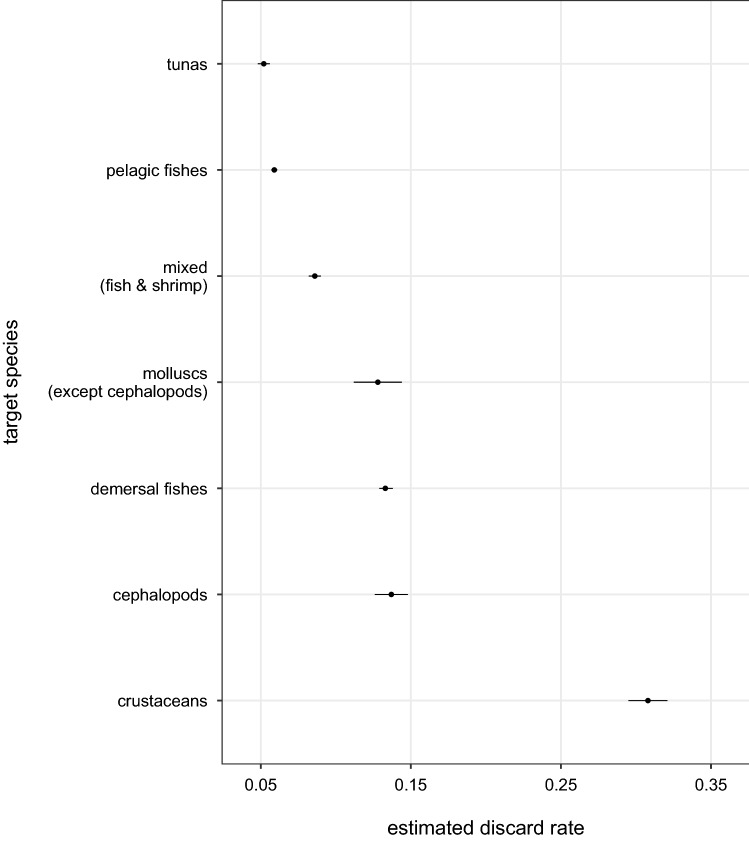
Mean discard rates and 95% HDIs by target species, in tonnes of discards per tonnes of total catch.

**Figure 5:**
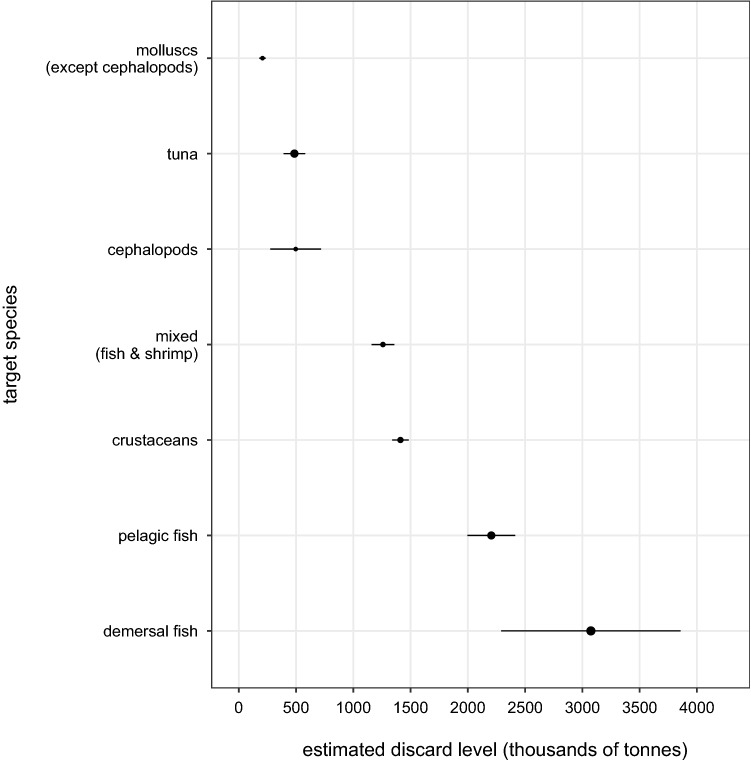
Mean discard levels in kilotonnes and 95% CIs by target species. The size of the circle for the mean is proportional to the sample size. Reproduced with permission from Perez Roda^24^.

Figures 4 and 5 present discard rates and levels by target species, respectively. Crustacean fisheries had the highest discard rate of 32.4% (95% HDI: 31.4–32.5) and tuna fisheries the lowest of 5.4% (95% HDI: 4.4–6.2). Fisheries targeting demersal fishes had the highest discard levels—contributing over a third of global discards, while fisheries targeting molluscs (excluding cephalopods) had the lowest—contributing only 2% of global levels.

## Discussion

FAO’s initial estimated discard level for the period 1988 to 1990 was about 27 million tonnes^26^, which was subsequently revised down to 20 million t^27^. Comparing this first FAO estimate to those of Kelleher^20^ for the period 1992–2005 and to the findings of the current study indicates a declining trend from the late 1980s to 2014, with the latest discard estimate being around half of the initial estimate. While this assessment of changes in estimated discard levels is compromised by different approaches being used in each of the three assessments, the temporal pattern and estimate from the current assessment are both consistent with the findings of Zeller et al.^7^. Zeller et al.^7^, accounting for estimated unreported catch, found that annual discards peaked at around 19 million t in 1989 and gradually declined to under 10 million t by 2014. Tropical shrimp, bottom trawl and other trawl gears, long identified as having relatively high discards^7,20^, remain the largest opportunities for further reductions.

The declines in discard levels and rates over the past few decades may have been due to: (i) the use of more selective fishing gear and methods that avoid the capture of unwanted species and sizes; (ii) increased retention of catches that had previously been discarded due to changes in market demand (e.g., species now used as feed by the aquaculture sector), and due to discard bans where effective; (iii) reduced effort by fisheries with relatively high discard rates; and (iv) reduced abundance of species that are discarded^6,7,20,28^. FAO^29^ hypothesized that a reduction in discards contributed to a 3.2% mean annual increase in global fish consumption that was observed to have occurred between 1961 and 2016.

We encountered several limitations that reduced the certainty of the discard estimates, including: small sample sizes of records of observed discard rates for some gear types (e.g., handline) and countries (e.g., East and Southeast Asian countries, Norway, Iceland), relying on expert judgement to allocate landings to individual fisheries by flag state, not attempting to account for unreported catch, assuming a linear relationship between discarded and total catch, and not assessing the species and size composition of discards. These limitations could be addressed in future studies. Furthermore, fishery-specific estimates of discard rates were not available for over two thirds of global fisheries. Addressing the low level or lack of observer coverage in the majority of global fisheries^11^, where electronic monitoring will be especially important in fisheries where conventional human observer coverage is problematic, would improve future discard estimates as well as help achieve other critical monitoring and management objectives. Periodic updated global discard assessments, with improvements in data quality and assessment approaches, are needed to assess the performance of discard reduction measures, where continued reductions will further reduce the wastage of natural resources and improve global food security.

## Methods

Discard rates and levels were estimated for global commercial marine and estuarine capture fisheries, by FAO area, gear type and target species. The weights of annual species-specific retained catches by individual country were obtained for the period 2010–2014 from the *FAO Global Fishery and Aquaculture Database*^30^ and *FAO Regional Capture Fisheries Database*^31^. Country-specific retained catch was allocated to 1,854 individual fisheries employing Kelleher’s^20^ definition of a fishery as determined by the flag state, gear type, target species and FAO fishing area. Discards were defined, consistent with Kelleher^20^, as the portion of the catch that is returned to the sea whole, alive or dead.

A new open access database was created with compiled records on retained and discard rates and levels by individual fishery^25^ (see Supplemental Information for details on the development of FAO’s *Discards Database for Global Marine Fisheries*, *25*). Fishery-specific discard rates were used for 419 fisheries for which records of discard rates were available. This covered 20% of global retained catch. Estimated discard rates were applied to 206 fisheries of 12 Asian countries and 2 countries with discard bans (Iceland and Norway), representing 45% of global retained catch (Supplemental Information Table S1). For the remaining 1,230 fisheries, representing 35% of global catch, global gear-specific discard rates were used. These global discard rates were estimated for 25 gear types with gear-specific zero-inflated Beta regression models^32^ fitted within a Bayesian inferential framework using the brms interface^33^ to the Stan computation engine^34^. Details on the model fitting and evaluation procedures used here can be found in Gilman et al.^35^, and details on the database of gear-specific discard rates is available in the Supplemental Information.

## Supplementary information

Supplementary Information.
