# Supplementary material for: Benchmarking global fisheries discards
Source: Sci Rep. 2020 Aug 20;10:14017. doi: 10.1038/s41598-020-71021-x (PMC7441149; doi:10.1038/s41598-020-71021-x)
Supplement: Supplementary file 1 — Supplementary Information. [file 41598_2020_71021_MOESM1_ESM.pdf]

Supplemental Information

Benchmarking global fisheries discards

Gilman, E., A. Perez Roda, T. Huntington, S. J. Kennelly, P. Suuronen, M. Chaloupka, P. A. H. Medley

Scientific Reports

This Supplemental Information provides additional information on the methods employed to develop the Food and Agriculture Organization of the United Nation's (FAO's) *Discards Database for Global Marine Fisheries* [1]. The weights of annual species-specific retained catches by flag state and FAO statistical were obtained for the period 2010-2014 from the *FAO Global Fishery and Aquaculture Database* [2] and *FAO Regional Capture Fisheries Database* [3]. Data on retained catch were obtained from the latter database for eastern central and southeast Atlantic regions. Retained catch weights by species were then allocated to 1,854 individual fisheries, where a fishery was defined based on the flag state, gear type, target species and FAO fishing area.

A literature review was conducted to compile records of discard rates. These compiled records were obtained from 156 compiled publications (the *DiscardRates* worksheet of FAO [1]), and provided fishery-specific discard rate estimates for 419 of the 1,854 global fisheries. The discard rate estimates were obtained from experiments, observer and logbook program data and stakeholder surveys, with a time series of 2000 to 2017. For 1,230 of the fisheries for which fishery-specific discard rates were not identified, global gear-specific discard rates were used, using the discard rate records compiled from the literature search, by employing the statistical modeling approach described in the Methods section of the article. Country-specific discard rates were applied to 206 fisheries of 12 countries (Table S1).

Table S1. Country-specific fisheries discard rates (tonnes of discards to tonnes of total catch).

Country	Discard rate	Citation
Bangladesh	0.063	4
Cambodia	0.007	5
China	0.050	6
Iceland	0.008	7
India	0.095	8
Indonesia	0.043	9
Korea, North	0.005	6
Malaysia	0.022	10
Myanmar	0.007	11
Norway	0.008	7
Philippines	0.007	12
Sri Lanka	0.095	8
Thailand	0.010	13
Vietnam	0.125	14

REFERENCES

1. FAO. *Discards Database for Global Marine Fisheries*. Available online, http://www.fao.org/fishery/static/TP633/datarepository.xlsm. Food and Agriculture Organization of the United Nations, Rome (2019)
2. FAO. *FAO Global Fishery and Aquaculture Dataset*. Version 2016.1.2. Food and Agriculture Organization of the United Nations, Rome (2016)
3. FAO. *FAO Regional Capture Fisheries Dataset*. Version 2016.1.0. Food and Agriculture Organization of the United Nations, Rome (2016)
4. Ullah, H., Gibson, D., Knip, D., Zylich, K..,& Zeller, D. Reconstruction of total marine fisheries catches for Bangladesh: 1950-2010. Fisheries Centre Working Paper #2014-15. University of British Columbia, Vancouver (2014)
5. Teh, L., Shon, S., Zylich, K., & Zeller, D. Reconstructing Cambodia's marine fisheries catch, 1950-2010. Fisheries Centre Working Paper #2014-18. University of British Columbia, Vancouver (2014)
6. Kelleher, K. *Discards in the World's Marine Fisheries. An Update*. FAO Fisheries Technical Paper No. 470. Food and Agriculture Organization of the United Nations, Rome (2005)
7. EU Data Collection Framework. FDI Data Call 2016. Landings, Value and Discards Data by Country. Finland and Iceland. Available online, https://stecf.jrc.ec.europa.eu/dd/bioeco/graphs, accessed 1 May 2016. Joint Research Centre of the European Commission, Ispra, Italy (2016)
8. Hornby, C., Bhathal, B., Pauly, D., & Zeller, D. Reconstruction of India's marine fish catch from 1950-2010. Fisheries Centre Working Paper #2015-77. University of British Columbia, Vancouver (2015)
9. Pauly, D., & Budimartono, V. Marine Fisheries Catches of Western, Central and Eastern Indonesia, 1950-2010. Fisheries Centre Working Paper #2015-61. University of British Columbia, Vancouver (2015)
10. Teh, L., & Teh, L. Reconstructing the marine fisheries catch of Peninsular Malaysia, Sarawak and Sabah, 1950-2010. Fisheries Centre Working Paper #2014-16. University of British Columbia, Vancouver (2014)
11. BOBLME. Fisheries catches for the Bay of Bengal Large Marine Ecosystem since 1950. BOBLME-2011-Ecology-16. Phuket, Thailand, Bay of Bengal Large Marine Ecosystem Project, Sea Around Us Project, Fisheries Centre, University of British Columbia, Vancouver, Canada (2011)
12. Palomares, M., & Pauly, D. Philippine Marine Fisheries Catches: A Bottom-up Reconstruction, 1950 to 2010. Fisheries Centre Research Report 22. University of British Columbia, Vancouver. (2014)
13. Derrick, B., Noranarttragoon, P., Zeller, D., The, L., & Pauly, D. Thailand's Missing Marine Fisheries Catch (1950–2014). *Front. Mar. Sci.* **4**,4 12 (2017)
14. The, L., Zeller, D., Zylich, K., Hguyen, G., & Harper, S. Reconstructing Vietnam's marine fisheries catch, 1950-2010. Fisheries Centre Working Paper #2014-17. University of British Columbia, Vancouver (2014)
